# Implementing and evaluating the German adaptation of the “Strengthening Families Program 10 - 14“– a randomized-controlled multicentre study

**DOI:** 10.1186/1471-2458-14-83

**Published:** 2014-01-27

**Authors:** Sonja Bröning, Peter-Michael Sack, Monika Thomsen, Martin Stolle, Astrid Wendell, Julian Stappenbeck, Rainer Thomasius

**Affiliations:** 1 Centre for Psychosocial Medicine, German Centre for Addiction Research in Childhood and Adolescence, University Medical Centre Hamburg-Eppendorf, Martinistraße 52, D-20246 Hamburg, Germany

**Keywords:** Family-based prevention, Preventive intervention, Manualised group programme, Substance use, Addiction, RCT

## Abstract

**Background:**

Substance use problems in childhood and adolescence can severely impact youth’s physical and mental well-being. When substance use is initiated early, the risk for moving from hazardous substance use to substance use disorders (SUD) is particularly high to developmentally induced biological and psychological vulnerability towards chronic trajectories in youth. Thus, risk factors for developing SUD should be addressed early in life by adequate preventive measures reaching out to children, adolescents, and their families. The study described in this protocol will test the effectiveness of the German adaptation of the *Strengthening Families Program for Parents and Youth 10–14 (SFP 10–14)* aimed at ten to 14 year old adolescents and their caregivers.

**Methods/Design:**

The study is conducted in four large German cities by counselling centres in the areas of youth welfare, social work and addiction aid. The effectiveness of the manualised group programme “Familien Stärken” consisting of seven sessions and four booster-sessions is tested among N = 288 children and participating parents in a multicentre randomised controlled trial with standardised assessment instruments. The control condition receives a minimal 2-hour intervention on parenting delivered in a school setting. Data are collected shortly before and after as well as six and 18 months after the intervention. We expect to replicate the favourable effects of the SFP 10–14 programme in the United States in the area of substance use initiation, family functioning and individual psychosocial adjustment.

**Discussion:**

The trial is expected to contribute to the growing literature on family-based preventive interventions, their effectiveness and feasibility. It is in line with several other current European efforts aimed at strengthening families against the detrimental effects of substance abuse in youth. The results of these trials will expand our knowledge on adapting evidence-based interventions and delivering them in diverse cultures and settings.

**Trial registration:**

Current Controlled Trials ISRCTN90251787

## Background

Substance use problems in childhood and adolescence severely impact physical and mental health and are often accompanied by developmental and comorbid mental disorders [1,2]. When they lead to SUD they cause high public costs that are predominantly the result of the protracted and difficult SUD treatment, of comorbidity, high relapse rates and the problematic (re-)integration into social life [3]. For children and adolescents, the risk for moving from hazardous substance use to SUD is particularly high due to developmentally induced biological and psychological vulnerability towards chronic trajectories. For instance, alcohol consumption impairs their developing learning and memory functions to a greater degree than it does in adults [4,5]. It also changes the reward system in that emotion regulation matures more slowly, while risks are perceived as less threatening. These effects evoke academic and social problems that in turn can result in further increased substance use. Studies show that the beginning of serious substance dependencies in adulthood often comes with early initiation of heavy substance use in late childhood or adolescence [6]. Early initiation and heavy use of substances are not the only factors predicting SUD development. The host of empirically demonstrated risk and protective factors influencing SUD trajectories includes genetic influences, individual differences such as gender, personality, mental health problems, emotional and social skills, social influences such as peer attitudes and behaviours as well as family influences, such as parental substance use, family relationships and environment [7-9].

Risk factors for developing SUD should be addressed early in life by adequate preventive measures reaching out to children, adolescents, and their families. Various prevention programmes have evolved and proved successful in reducing substance abuse and other behavioural problems in youth. Youth have been addressed in diverse settings, such as schools (e.g. Good Behaviour Game, [10]), communities, media and by approaching their parents with parenting advice (e.g. Triple P Positive Parenting Program, [11]). However, a growing body of literature suggests that interventions that address variables like family functioning, family communication patterns and parenting styles may be more effective [12]. One of the interventions that attained good outcome results is the Strengthening Families Program for Parents and Youth 10–14 (SFP 10–14) which was developed from 1993 on by Molgaard and colleagues at Iowa State University and has been studied for 25 years now. It was developed for the universal family-based prevention of addictive and behavioural problems in children and youth aged 10 to 14 years [13]. The programme is theoretically based on the socio-ecological model that emphasizes the relevance of the environment an organism develops in [14]. It focuses on the enhancement of family protective and resiliency processes and family risk reduction.

SFP 10–14 was evaluated in a longitudinal controlled study from 1993 to 2000 in rural economically deprived regions in Iowa [15-18]. The programme has consistently demonstrated favourable effects, even if their analysis has not remained without critique [19]. In a study with pre- and post intervention assessments and with follow-ups after 1.5, 2.5, 4 and 6 years, 446 families were surveyed with standardised questionnaires (238 families in the intervention group, 208 families serving as controls). Compared to controls, youth who had participated in SFP 10–14 showed a significantly lower average use of alcohol and tobacco after a 1-year period (with predominantly small effect sizes). In the 4-year follow-up they showed 30-day-prevalence that was 30% (alcohol) and 46% (tobacco) lower than prevalence in the control group. Furthermore, there was a significantly lower risk in the SFP group for using cannabis throughout life as well as in the 12-month-prevalence [17]. Self-reported aggressive conduct and behaviour as well as observer-rated aggressive and hostile behaviour in adolescent-parent interactions also were reduced compared to controls in the 4-year follow-up [20]. In the 6-year-follow-up, the favourable effects of the delay in substance use initiation for the SFP-group continued [18]. On average, the differences between intervention- and control group became more distinct over time (“sleeper effects”). Compared to controls, it was found that parents participating in SFP 10–14 [17] exhibited better parenting skills and more effective communication (expressing affection and setting limits) as well as better parental monitoring than parents in the control group.

To date, the German health care system severely lacks family-based preventive approaches. In order to make a well-established family-based prevention programme available to the German population, we translated SFP 10–14 and adapted it to German culture, taking into account family based interventions are especially culture-sensitive regarding role-model behaviour, values and norms [21]. The adaptation pilot study is described elsewhere in detail [22]. Our efforts link in with several other current European trials evaluating SFP 10–14 (Poland: [23]; United Kingdom: [21]; Spain: [24]; Italy: [25]). We now proceed to describe the evaluation study of the adapted programme, called *Familien stärken.*

## Methods/Design

Our study is funded by the German Federal Ministry of Education and Research (BMBF; September 2009 – January 2014; grant 01EL0810). It is guided by two central research questions: (1) is the adapted German programme version effective and how does its effectiveness compare with the US-based findings as well as the findings from current EU trials? (2) Does programme effectiveness vary between subgroups within the sample (e. g., families with single parents, families with high psychopathology prior the intervention) and what does this tell us about the working mechanisms of the intervention? To answer these questions, we are currently conducting a randomised-controlled multicentre study with assessments scheduled before (pre, t0) and after (post, t1) the intervention, as well as follow-up assessments 6 (t2) respectively 18 months (t3) after the intervention has ended. Except for SES, only standardised instruments are employed; see assessment paragraph below for details. The study is conducted in four large German cities (Hamburg, Schwerin, Hanover and Munich). Families are mainly recruited in schools by staff from the local cooperating counselling centres in the areas of youth welfare, social work and addiction aid. To get in touch with families, counsellors will attend parent’s evenings to inform parents and caretakers about the project and hand out brochures. Parents who are interested in the programme are invited to an introductory appointment at which they are informed in detail about study procedures, and then give informed consent if they are willing to participate in the study. In order to reach families with low socio-economic status (SES), recruitment will only take place in socially deprived urban districts. Districts are defined as deprived if the percentage of inhabitants who are eligible for social security benefits is significantly higher (one standard deviation) than it is for the general population in that city. Another measure to attain the low SES sample we are aiming at is to limit the percentage of children who attend secondary school to 30%.

Families are admitted to the study if they have at least one child aged 10–14 years and at least one parent that is willing and able to participate in the group programme. Families are excluded if the child has a manifest substance use disorder or displays serious behavioural problems that would interfere with the group format. Children who are currently or were in the past six months enrolled in a group programme with similar goals are also excluded. In each city, 72 families are to be recruited half of which are randomly assigned to the intervention group, while the other half constitutes the control group. Thus, we aim for a total of N = 288 participating families at baseline. This sample sizes is required [26] for detecting at least medium effect sizes in 2-point repeated measurements (like t0-t1), given a two-sided test at alpha = 0.05 and a power of 1–beta = 0.80. It is large enough to also detect small effect sizes in 4-point repeated measurements (t0-t1-t2-t3). It already takes a worst case scenario of 33% loss-to-follow-up after randomisation into account, so N = 288 is the sample sizes needed at t0. Randomisation is conducted by first matching pairs according to the criteria of school site (location of attended school), school type (educational level), sex, age (in this order) and then allocating them randomly to one of the two groups.

### Intervention and training

Familien stärken is the approved German adaptation of the Iowa SFP 10–14. Like SFP 10–14, it is manual-based and consists of seven weekly sessions plus another four booster sessions that are conducted 4–6 months after the seventh session ends. In each session, at least three group facilitators work with 8–12 families. Programme elements are tailored to adequately address parents, children, and the entire family, respectively. SFP 10–14 aims at reducing the risk for SUD and behavioural problems. In the first part, youth and their parents meet in parallel separate sessions. Youth modules aim at improving youth’s self-efficacy and their ability to cope with stress and peer pressure. Parent modules encourage caregivers to reflect their parenting style, to develop a more consistent form of parenting (“using love and limits”) and to express positive affect more openly. In the ensuing family sessions, dysfunctional communication patterns within the family are addressed and family cohesiveness is strengthened. Special care is taken to secure high retention rates during programme participation. After each meeting, families are invited for a complementary meal which is either served by external caterers or affiliated restaurants. Also, child care is offered for families who have smaller siblings they cannot leave at home alone. As an additional incentive, families receive a voucher worth € 15 after every session. It is up to the agencies what kind of vouchers they offer; examples are cinema or swimming baths vouchers.

Facilitators were recruited among the staff of the four cooperating agencies. Most of them have a background in social work as well as several years of working experience in the field of family and youth counselling. A total of 21 facilitators were trained by experienced master trainers from the UK (Oxford Brooks University). During this mandatory three day training in Hamburg, Germany, the entire programme was presented, introducing its key elements and providing future facilitators with the opportunity to experience programme activities as participants, but also as trainers. The first author of this paper worked as an interpreter for the master trainers.

We developed a minimal intervention to control for assessment reactivity effects. The programme gives information about the physical and mental changes affecting teenage youth. Parents are informed how they best can react to these changes and keep a trusting relationship. Video segments are used to show typical conflict situations at home and different ways to react to them. Material for this intervention came from a brochure with information about parenting for parents of adolescents (Starke Kinder - Ein Magazin für Eltern, BZgA; http://www.bzga.org), while the video segments came from another video-based prevention programme (“Freiheit in Grenzen”, [27]). A presentation with instructions along with video segments was sent to the cooperating agencies. At each agency, a staff member was assigned to deliver the event adhering closely to the slide show and instructions. Families allocated to the control condition are invited to a one-evening-only event, at which the two-hour programme was delivered. The event is closed with a complementary meal.

### Assessment

Two versions of a test battery were created, one for parents and caretakers, and another one for youth. We assess SES, tobacco, alcohol and illicit drug use, mental health indicators, and a variety of psychosocial constructs such as self-efficacy. Both test batteries also contain several questionnaires on family functioning. A self-constructed questionnaire is used for SES assessment, all other instruments are standardised and validated. Instruments are given in Table 1. Children’s substance use prevalence is retrieved by self-reports and validated by urinalyses. Tobacco consumption can be detected via cotinine (3–4 days), alcohol via ethylglucuronide (3–4 days) and cannabis via THC-metabolite (1–6 weeks). Even though short half-lives prevent the exclusion of intermediate tobacco or alcohol intake, regular consumption patterns can be detected. At baseline, and in order to minimize youth resistance to urine screening, parents and children consent to the fact that parents will not be informed on urinalysis results. Table 1 gives an overview of the measures used in our study.

**Table 1:** Assessment measures used in the German evaluation trial of SFP 10-14

**Variable**	**Instrument**	**Who is assessed?**	**Measuring point**
Personal data	SES (Socio-economic status) Self constructed questionnaire	Parents	t0
Substance use assessment	Structured Interview/urinalysis	Youth	t0 – t3
Screening of mental problems	BSI (Brief Symptom Inventory) [28,29]	Parents	t0 – t3
Screening of mental problems	SPS-J/RAASI (Screening for psychological Symptoms in Youth) [30,31]	Youth	t0 – t3
Behavioural problems	SDQ (Strengths and Difficulties Questionnaire) [32,33]	Youth/Parents	t0 – t3
Attitudes toward drug use	Substance Use Affinities in Youth [34]	Youth	t0 – t3
Quality of life	ILK (Inventory of Life Quality in Children and Adolescents) [35]	Youth	t0 – t3
Self-efficacy	SWE (General Self-efficacy Scale) [36]	Youth/Parents	t0 – t3
Parenting style	ESI (Parenting Style Inventory) [37]	Youth	t0 – t3
Family functioning	FACES IV (Family Adaptability and Cohesion Evaluation Scales) [38], own translation	Youth/Parents	t0 – t3
Family functioning	FAM (Family Assessment Measure) [39]	Youth/Parents	t0 – t3
Programme Satisfaction	FBB (Treatment Satisfaction Questionnaire) [40]	Youth/Parents	t0 – t3

As measure to decrease dropout rates, trained research staff members visit the families in their homes and hand out the questionnaire forms. Youth and parents answer their questionnaires in separate rooms, and staff members check questionnaires for completeness. Youth’s urine samples are taken at the families’ homes and shipped to a toxicological institute (Department of Legal Medicine of the University Medical Centre, Hamburg) for analysis. Control families are paid € 50 for each assessment, while intervention families receive incentives only for the last two assessments (due to the other incentives they receive during programme participation). For process evaluation purposes, trainers are asked to report on their professional background and work experience. After each training session, trainers report on whether or not they had covered the sessions’ key activities. Youth and parents also are requested to evaluate the programme after the last session. Sessions 4 and 5 are videotaped to enhance programme fidelity and enable intervention competence analyses.

### Measures against bias

At t0, research staff members who collect data in families’ homes are unaware of the families’ study condition. For the other assessments they are informed about condition status due to practical reasons (the control group parents are paid incentives for the t1-interview). As mentioned above, families are balanced between conditions regarding school site, school type, sex and age. In statistical analyses, control measures like baseline-adjusted testing, drop-out and ITT analyses will be employed.

### Ethical considerations

All procedures were approved by the ethics committee of the Chamber of Physicians of Hamburg, Germany. All participants of the study are informed about the study goals, procedures, analyses and data reporting prior to participation. Only data of participants (parents and youth) who gave written consent will be used for analysis. In recording video tapes for fidelity purposes, we make sure that only trainers are visible, not programme participants. Participant data are collected pseudonymised, while data analyses and published material will be anonymised allowing no conclusions on individual study participants. All data treatment is implemented according to national data security law.

### Analysis

Socio-demographic data like age, sex, school type and net household income will be compared between intervention and control group in order to check if matching of families had been successful. Longitudinal as well as cross-sectional analyses are planned as soon as post-intervention data are available, including ITT analyses. Preferred methods to test effectiveness will be repeated measures baseline-adjusted ANCOVAs, and Mixed Models to further investigate the relationships between certain characteristics of the sample and programme effectiveness. Depending on data structure, Growth Curve Analysis or Multilevel Analyses will be used to estimate and predict subjects’ development regarding substance use and behaviour problems in the future. Correlations between programme adherence, intervention competence and outcome will be applied to test for possible relations of the three variables.

## Discussion

This is the first study to evaluate a family-based SUD prevention programme in Germany. The internationally established and reputed Strengthening Families Program 10–14 and its adapted German version Familien stärken are tested in a methodologically extensive and thorough analysis. This will lead to findings that answer various questions: First, it will become clear whether German adaptation of the SFP 10–14 shows effective results, can be delivered in German ambulant health care settings, and is well-received by parents, youth and trainers. In addition to these findings, important results are expected from subgroup analyses: Are there types of families that benefit more than others from the training? Are there differences between families who drop out of the study compared to those who stay? Empirical answers to these questions will inform future development and refinement of prevention programmes for youth and their families. In sum, the trial is expected to contribute to the growing literature on family-based preventive interventions. It is in line with several other current European efforts aimed at strengthening families against the detrimental effects of substance abuse in youth. The results of these trials will expand our knowledge on the effectiveness of family-based prevention and on adapting evidence-based interventions for diverse cultures and settings.

## Competing interests

The authors declare that they have no competing interests.

## Authors’ contributions

SB and JS drafted the manuscript. MS, PMS, and RT obtained funding. SB and MT coordinate the study, while JS and AW participated substantially in designing the intervention and study methodology. PMS is responsible for data analysis. RT was involved in all parts of the article as general supervisor of the research group. All authors have read and approved the final manuscript.

## Authors’ information

The authors of this paper work at the German Center for Addiction Research in Childhood and Adolescence (Deutsches Zentrum für Suchtfragen des Kindes- und Jugendalters; DZSKJ) currently conducting this national trial of the German adaptation of the Strengthening Families Program 1014 in Germany (http://www.familienstaerken.info). For more information on our research, see http://www.dzskj.de.

## Pre-publication history

The pre-publication history for this paper can be accessed here:

http://www.biomedcentral.com/1471-2458/14/83/prepub
